# Thromboinflammatory Biomarkers Are Early Predictors of Disease Progression in Non-Small Cell Lung Cancer Patients

**DOI:** 10.3390/cancers17121932

**Published:** 2025-06-10

**Authors:** Patricia Gomez-Rosas, Carmen Julia Tartari, Laura Russo, Silvia Bolognini, Chiara Ticozzi, Debora Romeo, Francesca Schieppati, Luca Barcella, Anna Falanga, Marina Marchetti

**Affiliations:** 1 Immunohematology and Transfusion Medicine, Hospital Papa Giovanni XXIII, 24127 Bergamo, Italy; patmlhs@hotmail.com (P.G.-R.); annafalanga@yahoo.com (A.F.); 2 Cardiovascular Research Institute Maastricht (CARIM), Maastricht University Medical Center (MUMC+), 6229 ER Maastricht, The Netherlands; 3 Hospital de Oncologia, Unidad Medica de Alta Especialidad (UMAE), Centro Medico Nacional Siglo XXI, Instituto Mexicano del Seguro Social (IMSS), Mexico City 06720, Mexico; 4 School of Medicine and Surgery, University of Milan Bicocca, 20126 Milan, Italy

**Keywords:** cancer-progression, non-small cell lung cancer-disease progression, high-sensitivity C-reactive protein, d-dimer, thromboinflammation

## Abstract

(1) Background: The hemostatic system and tumor biology display a tight and reciprocal interaction where clotting products enhance tumor growth and dissemination, and the tumor, in turn, triggers a hypercoagulable and inflammatory state. Evaluating circulating biomarkers related to thrombo-inflammatory may provide a promising tool for predicting tumor outcomes, especially in non-small cell lung cancer (NSCLC) characterized by unfavorable outcomes. (2) Aim: In a prospective cohort of NSCLC patients, we evaluated whether thromboinflammatory biomarkers could predict early disease progression (DP) during the first 6 months of first-line anticancer treatment. (3) Methods: 719 newly diagnosed advanced-stage NSCLC patients were included. Complete blood cell count, high-sensitivity C-reactive protein (hs-CRP), FVIII, fibrinogen, D-dimer, thrombin-antithrombin (TAT) complexes, and prothrombin fragment1+2(F1+2) were tested in blood samples collected before starting chemotherapy. DP was gathered during follow-up. (4) Results: The 6-month cumulative incidence rate for DP was 49%. Univariable Cox regression analysis identified metastatic status, BMI, hemoglobin, leukocytes, hs-CRP, FVIII, fibrinogen, TAT, and D-dimer as significant predictors of DP. In a multivariable analysis that included all previously significant variables, only hs-CRP and D-Dimer levels remained strongly associated with DP. The two variables were used to establish a risk stratification model that significantly identified patients at high risk of DP at 6 months (HR 2.9; 95% CI, 2.3–3.7), which can be applied to 3, 9, and 12 months. (5) Conclusions: Our model easily and precisely estimates early DP during chemotherapy. If externally validated, this model can significantly enhance the allocation of medical resources in managing advanced NSCLC, ensuring that patients receive the most effective care possible.

## Simple Summary

Non-small cell lung cancer (NSCLC) presents a significant challenge due to its poor prognosis, highlighting the need for innovative predictive tools to improve patient outcomes. In a prospective cohort study involving patients with newly diagnosed NSCLC at various stages, we conducted a thorough evaluation of clinical and tumor characteristics, along with a panel of thromboinflammatory biomarkers, prior to the initiation of cancer treatment. We aimed to assess the potential of these factors in predicting cancer progression. Our findings show that high-sensitivity C-reactive protein and D-dimer are strong predictors of disease progression. Based on these biomarkers, we developed an easy-to-apply model that allows us to identify patients at a three-fold higher risk of early disease progression.

## 1. Introduction

Lung cancer represents the leading cause of cancer-related mortality globally, accounting for about 1.8 million deaths each year [1]. This aggressive malignancy is frequently associated with a poor prognosis, reflected in an overall 5-year survival rate of approximately 25% [2].

The most important prognostic factors in NSCLC include clinical factors such as disease stage and performance status, biological factors such as genetic mutations and expression of biomarkers, and therapeutic factors related to response to treatments and therapeutic modalities adopted [3,4,5]. Other pathologies, such as cardiovascular or pulmonary diseases, also adversely affect the prognosis [6,7]. Patients with comorbidities have a reduced tolerance to treatments and a lower quality of life. Unfortunately, many cases of NSCLC are diagnosed at later stages when the cancer has already spread, making treatment more difficult and reducing the chances of a favorable outcome [8,9]. The TNM staging system (Tumor, Node, Metastasis) is fundamental for determining the prognosis of NSCLC; however, it does have some limitations [10]. Patients at the same stage can experience significantly different outcomes, as the staging system does not fully account for the complex interplay of various factors.

This underscores the critical need for improved methods to predict patient outcomes or prognostic tools in NSCLC. A key area of investigation focuses on the intricate relationship between the hemostatic system and the biology of the tumor itself [11]. This relationship is characterized by a two-way interaction: components of the clotting system can promote tumor growth and spread (metastasis), while the tumor can induce a hypercoagulable state and a pro-inflammatory microenvironment [12,13,14]. Coagulation and inflammation are interconnected processes. Inflammation can initiate the coagulation cascade, while the activation of coagulation can enhance inflammation. These interactions can create feedback loops that sustain both coagulation and inflammation, which may contribute to the development and progression of cancer. Indeed, the occurrence of a venous thromboembolic event (VTE) significantly raises the risk of early mortality in cancer patients undergoing chemotherapy. It is also associated with a greater likelihood of tumor progression and reduced long-term survival [15,16]. Consequently, several published risk assessment models (RAMs) for VTE during chemotherapy have been shown to predict mortality and progression as well [17,18,19].

Given the strong relationship between inflammation, coagulation, and cancer, measuring levels of specific plasma biomarkers as surrogates of in vivo activation of hemostatic and inflammatory pathways can be a valuable tool for evaluating different outcomes in individual patients. Numerous studies have investigated systemic inflammation biomarkers and shown their significant prognostic value across different cancers, including NSCLC [20,21,22,23,24]. However, the optimal combination of inflammatory and hemostatic parameters for predicting prognosis in NSCLC patients remains to be determined.

The current study aimed to evaluate whether baseline inflammatory and hemostatic biomarkers could predict the prognosis of patients with newly diagnosed advanced NSCLC in a prospective multicenter cohort. We specifically examined the feasibility of developing a predictive model that combines hemostatic and inflammatory biomarkers to serve as a prognostic tool for these patients. Prognostic factors help predict patient outcomes regardless of treatment. In contrast, predictive factors assess the outcomes of patients receiving specific systemic therapies and are closely associated with sensitivity or resistance to those therapies. We focused on predicting early disease progression (DP) within the first 6 months of first-line chemotherapy. Predicting tumor progression is valuable for improving cancer management, personalizing treatment, enhancing outcomes, reducing toxicity, optimizing resources, and fostering research.

## 2. Materials and Methods

### 2.1. Study Design and Subjects

Newly diagnosed, advanced-stage NSCLC patients (locally advanced and metastatic) were included. The patients have been enrolled in the framework of the Italian prospective, observational multicenter HYPERCAN study (Clinical Trials.gov ID# NCT02622815). The study’s design, methods, and development were previously described [25]. Briefly, clinical data, and blood samples were collected from participants before the initiation of chemotherapy, and they were prospectively followed for up to 5 years to assess various outcomes. The inclusion criteria consisted of adult patients (age ≥ 18 years) with a new diagnosis of advanced-stage NSCLC (metastatic stage TXNXM1 or locally advanced beyond stages IIIA, IIIB, or IIIC, characterized by T3–T4 and N2–N3) before starting anticancer treatment. Patients must have a life expectancy greater than 3 months and be candidates for first-line systemic chemotherapy. Age, gender, body mass index (BMI), Eastern Cooperative Oncology Group (ECOG) performance status, comorbidities, prophylactic use of anticoagulants (for any reason other than cancer), use of antiplatelet drugs, tumor histological subtype, and tumor biological characteristics were recorded at enrollment. After inclusion, patients were followed for a minimum of five years, and clinical data concerning antitumor treatment, clinical response, and mortality within 3, 6, and 12 months were evaluated by the treating physician. Exclusion criteria included any acute medical illnesses, hospitalization, or therapeutic anticoagulation at the time of enrollment.

Patients were enrolled between May 2012 and July 2020, and all events (DP and death) were validated by the Independent Central Adjudication Committee for inclusion in the analysis. For metastatic and locally advanced patients not candidates for surgical resection, the Response Evaluation Criteria in Solid Tumors, version 1.1 (RECIST 1.1) [26], was assessed after three and six cycles of chemotherapy. For locally advanced patients who underwent preoperative treatment followed by radical resection, the RECIST evaluation was performed approximately two weeks after the last chemotherapy, as part of the protocol for potentially resectable tumors. Written informed consent was obtained from all participating patients. The study was approved by all participating institutions’ ethics committees and conducted following the last revision of the Helsinki Declaration.

### 2.2. Blood Withdrawal and Processing

Peripheral venous blood was collected into no-additive tubes (BD Vacutainer^®^ SST™ II, Becton, Dickinson, Franklin Lakes, NJ, USA) and 6 mL vacutainer tubes containing 0.109 M sodium citrate (9:1 *v*/*v*; Becton Dickinson, Vacutainer, Plymouth, UK). Within 2 h of blood collection, serum was separated using a one-step centrifugation (3000× *g* for 10 min). A two-step centrifugation of 3000× *g* for 10 min at room temperature of citrated blood was performed to obtain platelet-free plasma. Aliquots of serum and plasma were then obtained and stored in liquid nitrogen. The procedures for obtaining, managing, and storing blood samples were carried out according to international standards [27].

### 2.3. High-Sensitivity C-Reactive Protein Analysis

A commercial solid-phase, two-step capture enzyme-linked immunosorbent assay (ELISA) was used to measure serum levels of high-sensitivity C-reactive protein (hs-CRP), according to the manufacturer’s instructions (Labor Diagnostika Nord, Germany, LDNTM). In brief, serum samples and an anti-CRP-Horseradish Peroxidase (HRP) conjugate were added to wells coated with a polyclonal antibody to CRP. Any unbound proteins and excess HRP conjugate were subsequently washed away. A standard curve was established using the manufacturer’s standards. The absorbance was measured at 450 nm using an ultra-microplate reader (Thermo Scientific™ Multiskan™ FC, Thermo Fisher Scientific Inc., Waltham, MA, USA).

### 2.4. Hemostatic Biomarkers Analysis

A panel of hemostatic biomarkers was tested in the plasma samples. Factor VIII (FVIII) was measured using the HemosIL FVIII:c assay (Werfen, Milan, Italy), and fibrinogen levels were assessed with the QFA thrombin assay (Werfen). Both tests were conducted on an automated coagulometer analyzer (ACL TOP500, Werfen Group, Milan, Italy). D-dimer levels were measured using the STA Liatest D-Di PLUS on the STA Compact Max 3 coagulation analyzer, following the manufacturer’s procedures. Thrombin-antithrombin (TAT) complex was measured using a homemade ELISA, as previously described [28]. Prothrombin fragment 1+2 (F1+2) levels were assessed using a commercially available ELISA (Enzygnost^®^, Siemens Healthcare Diagnostics, Munich, Germany), with an absorbance measured at 450 nm (Thermo Scientific™ Multiskan™ FC).

### 2.5. Outcomes

This primary outcome was DP development within six months of enrollment. The outcome was provided by treatment oncologist experts who also estimated disease control rate (DCR), classified as complete response (CR), partial response (OR), and stable disease (SD), according to RECIST1.1. The secondary outcome was DP within 3, 9, and 12 months from enrollment.

### 2.6. Statistical Analysis

Clinical characteristics were summarized as frequencies and percentages, and continuous variables as median with interquartile range (IQR) or mean with standard deviation (SD). Normally and non-normally distributed quantitative data were compared using the unpaired Student’s *t*-test and Mann–Whitney U test, respectively, while the chi-squared test was used to compare categorical data. The significance level was set at <0.05. Univariable Cox proportional hazard regression (HR) models (stcox STATA) were employed to assess the predictive value of clinical and laboratory variables concerning the endpoint of interest. All significant (*p* < 0.05) clinical and laboratory variables identified in the univariable analysis were included in the multivariable Cox model, using a forward likelihood estimates variable selection algorithm. A condition index test was applied to exclude multicollinearity. The predictive accuracy of the thromboinflammatory biomarkers-based model was assessed using C-statistics and plotted by the receiver operating characteristic (ROC) curve. A 1000 bootstrap-based optimism correction method was used to assess the model’s predictive ability as an internal validation, and a five-fold multiple imputation technique was employed to address missing data, thereby yielding less biased estimates of the score. The Kaplan–Meier method estimated the cumulative events of DP, with follow-up time restricted to 3, 6, 9, and 12 months, and assessed inter-group differences using the log-rank test. In case of surgery before the RECIST evaluation, patients were censored. A restricted cubic spline with 4 knots (RCS, mkspline STATA) curve was used to explore the association between the model and the outcome. A calibration plot was also applied to evaluate the model’s performance based on the observed and predicted event (pmcalplot, STATA), in accordance with TRIPOD guidelines [29]. The statistical analysis was performed using StataCorp. 2019. Stata Statistical Software: Release 19 (StataCorp LLC, College Station, TX, USA).

## 3. Results

### 3.1. Baseline Characteristics of the Study Population

**Figure 1:**
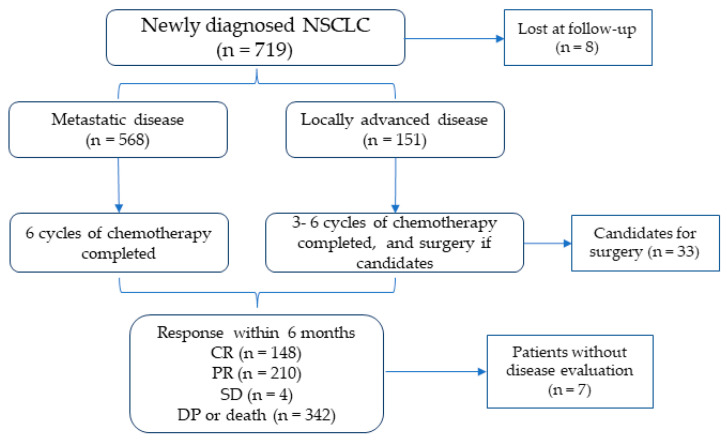
Study cohort flow chart. NSCLC: non-small cell lung cancer; CR: complete response; PR: partial response; SD: stable disease; DP: disease progression.

The study cohort, depicted in Figure 1, consisted of 719 patients diagnosed with advanced-stage NSCLC. A total of 8 patients were lost to follow-up during the study period, and in 7 patients, the outcome was not evaluated, resulting in a final cohort of 704 patients. At enrollment, 79% of the patients had metastatic disease (TXNXM1), while 21% exhibited locally advanced disease (beyond stages IIIA, IIIB, or IIIC (T3–T4 and N2–N3)). After enrollment, all patients started antitumor therapies, which were mainly based on chemotherapy. Patients with metastatic disease received six cycles of chemotherapy, whereas those with locally advanced disease candidates for tumor resection underwent three cycles of chemotherapy before surgical intervention.

### 3.2. Study Outcome

At the six-month follow-up, 342 patients experienced DP, resulting in a cumulative incidence of 49% (95% CI, 47–54) with a median time to occurrence of 136 days (95% CI, 132–140). During the same period, 183 deaths were recorded, all attributed to DP and one to a fatal pulmonary embolism, providing a cumulative incidence of mortality of 27% (95% CI, 13–41). The median time to death was 108 days (95% CI 66–145). In patients who achieved DCR at 6 months, the response to treatment was a CR in 41%, a PR in 58%, and a SD in 1%.

### 3.3. Characteristics of Patients According to DP

The clinical and histological characteristics of patients according to DP are detailed in Table 1. A significantly higher rate of patients who progressed within 6 months had metastatic disease (87% vs. 73%, *p* < 0.01). Furthermore, patients who progressed were characterized by poorer ECOG performance status (*p* < 0.001), higher leukocyte and platelet counts (*p* = 0.001), and lower hemoglobin and hematocrit levels (*p* = 0.042) compared to those who achieved DCR. Despite being slightly older than those who did not develop DP, the difference was not statistically significant (*p* = 0.066). No significant differences were observed in the rates of the different antitumor regimens between the two groups. A total of 33 patients from the locally advanced group underwent surgery in a median time of 102 days (IQR 89–117) from enrollment. Radiotherapy, specifically radical radiotherapy, was more frequently prescribed in patients who achieved DCR than in DP patients (*p* < 0.001).

### 3.4. Inflammatory and Hemostatic Biomarker Distribution

**Figure 2:**
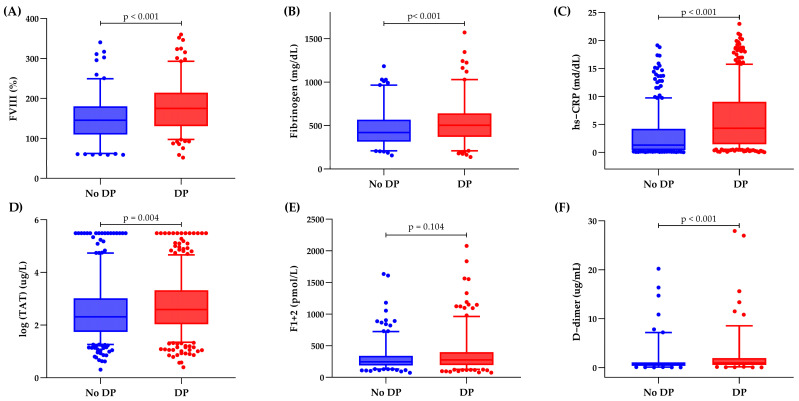
Distribution of thromboinflammatory biomarkers according to DP and DCR. (**A**) Factor VIII, (**B**) Fibrinogen, (**C**) high-sensitivity C-reactive protein, (**D**) Thrombin-Antithrombin complex, (**E**) Prothrombin fragment 1+2, (**F**) D-dimer.

Figure 2 shows the inflammatory and hemostatic biomarker distribution at enrollment according to DP and DCR at 6 months. Patients who developed DP demonstrated significantly (*p* < 0.004) elevated baseline levels of various laboratory markers, including hs-CRP, FVIII, fibrinogen, TAT, and D-dimer, compared to patients who achieved DCR. Patients who achieved a CR had the lowest baseline median levels of hs-CRP, FVIII, fibrinogen, and D-dimer compared to those who progressed. Additionally, patients with a PR exhibited lower levels of hs-CRP, FVIII, and D-dimer than those with a 6-month DP. No differences in baseline biomarker levels were found between patients with a CR and those with a PR (Supplemental Figure S1).

### 3.5. Clinical and Thromboinflammatory Biomarkers and Risk of DP

A univariable Cox regression analysis was performed on the baseline clinical and laboratory variables to identify predictors of DP (Table 2). Metastatic status and lower BMI at enrollment were significant (*p* < 0.05) independent clinical predictors of 6-month DP, while radiotherapy emerged as a protective factor (*p* < 0.001). Among laboratory variables, baseline higher levels of leukocytes, hs-CRP, FVIII, fibrinogen, TAT, and D-dimer, and lower hemoglobin levels were significant (*p* = 0.011) independent predictors of DP. However, only hs-CRP and D-dimer levels remained significantly associated with DP in the multivariable analysis, based on a forward likelihood algorithm that included the clinical and laboratory significant variables identified in the univariable analysis (Table 2). The condition index of the two predictive variables yielded a value of 2.509, thus excluding significant collinearity.

### 3.6. DP Modeling at 6 Months

Based on the alpha value provided by the multivariate analysis, D-dimer and hs-CRP were selected to generate the prognostic model for DP. First, we established ranges of hs-CRP levels based on the American Heart Association recommendation (i.e., <1.0, 1.0–3.0, >3.0 mg/L) [30], and for D-dimer, based on those utilized for the HYPERCAN-VTE score (i.e., <0.5, 0.5–1.5, >1.5–4, >4 µg/mL) [31]. Points were assigned to these hs-CRP and D-dimer ranges, as shown in Table 3. The points were summed, and cut-off values were identified based on the IQR of the sum to stratify the risk of DP according to three categories: ≤ 2 points = low, 3–4 = intermediate, and 5–6 = high-risk.

**Figure 3:**
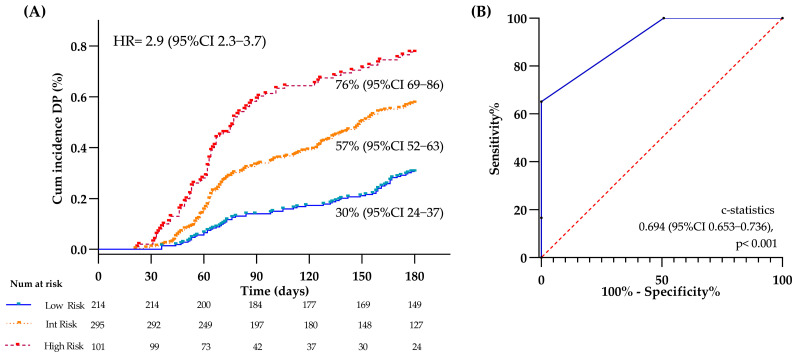
Cumulative incidence of 6-month DP and accuracy of the model. (**A**) Cumulative incidence of disease progression (DP) at six months stratified at low-, intermediate (inter)-, and high-risk. (**B**) Receiver operating characteristic (ROC) curve of the model’s predictive accuracy for DP.

Figure 3 shows the cumulative incidence of DP and the model’s accuracy for DP. Specifically, the 6-month cumulative incidence of DP was 30%, 57%, and 76% for the low, intermediate, and high-risk groups, respectively. By a 1000 bootstrap-based model correction, the risk of DP achieved a HR of 2.906 (95% CI 2.297–3.676), log-rank < 0.001 (Figure 3A). The ROC curve showed an AUC of 0.694, *p* < 0.001 (Figure 3B). After conducting multiple imputation analyses, the risk of DP achieved a HR of 2.884 (95% CI 2.192–3.790), *p* < 0.001, and an AUC of 0.687 (0.648–0.726).

**Figure 4:**
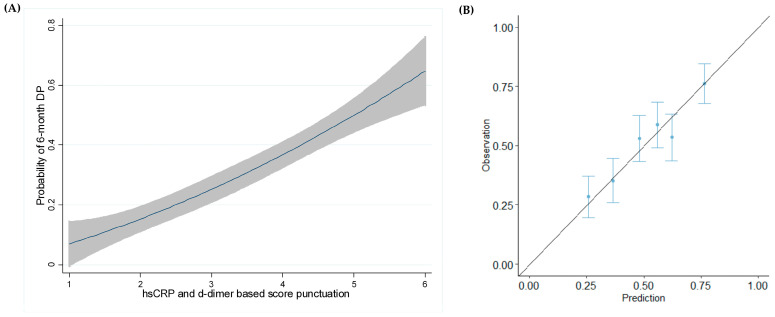
(**A**) Cubic 4-knot spline and (**B**) calibration plot of the model.

In addition, a four-knot restricted cubic spline analysis was performed to rule out potential nonlinear associations in the model in which DP risk increased with a higher punctuation of the score (Figure 4A). The calibration plot demonstrated the reliability of the model (Figure 4B).

### 3.7. Evaluation of the Model at Different Time Points

**Figure 5:**
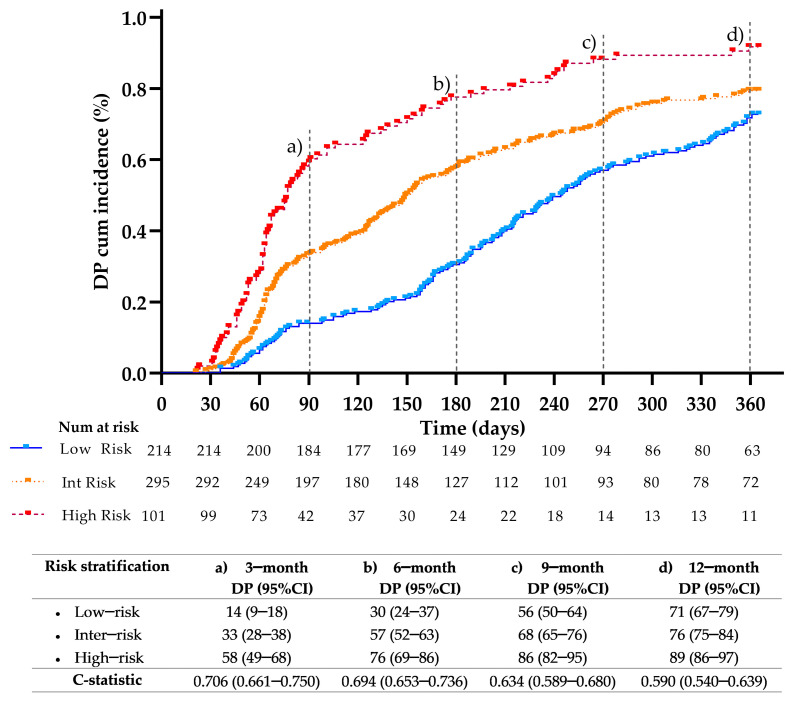
DP cumulative incidence of the model applied to different timeframes.

We then evaluated whether the model could accurately stratify patients at time points other than 6 months. Figure 5 shows the stratification rates of DP at 3, 6, 9, and 12 months as well as the model’s accuracy across different cut-off times. The results indicated a steady rise in DP rates across risk groups. At 3 months, the DP rates were 14% in the low-risk group, 33% in the intermediate-risk group, and 58% in the high-risk group. By 6 months, these rates increased to 30%, 57%, and 76%, respectively. At 9 months, they reached 56% for the low-risk, 68% for the intermediate-risk, and 86% for the high-risk group. Finally, at 12 months, the rates were 71%, 76%, and 89%, respectively. The model’s accuracy, measured by c-statistics, was highest at 3 and 6 months, with values of 0.706 and 0.694, respectively, compared to 9 months (0.634) and 12 months (0.590). The model was also applied to evaluate its prognostic value among the sub-cohorts of metastatic and locally advanced patients, as well as in the group of patients receiving contemporary treatments (i.e., immune checkpoint inhibitors or targeted therapies). As indicated in Supplemental Table S1, the model significantly predicted the development of DP across all sub-cohorts at 3, 6, 9, and 12 months, except for the group receiving contemporary treatments, for which the model did not reach significance at 3 months (*p* = 0.058). Finally, the model, regenerated after excluding subjects who underwent radical radiotherapy and surgery, was also able to significantly (*p* < 0.001) stratify patients into the three risk categories at different time points, as it did in the entire cohort. Specifically, the cumulative incidence of DP was 33% in the low-risk group, 60% in the intermediate group, and 76% in the high-risk group, with HR 2.003, log-rank *p* < 0.001, and a c-statistics of 0.673, *p* < 0.001.

### 3.8. Relationship of VTE with DP

Given the impact of thromboinflammatory biomarkers on DP, we assessed the prevalence of VTE in patients who experienced progression. A total of 68 VTEs were recorded within six months, 42 in patients who developed DP and 26 in those who achieved a DCR (chi-squared = 0.028). Among the 33 patients who underwent cancer surgery, no VTE were documented in the perioperative period until the end of follow-up. The occurrence of VTE within the six months from enrollment was significantly associated with a greater likelihood of developing DP at 12 months compared to those without VTE (HR 1.352; 95% CI, 1.018–1.794, *p* = 0.037). Published RAMs designed for VTE were tested in our cohort to determine whether they could predict DP (Supplemental Table S2). We found that patients identified as high VTE risk by models such as the KRS (HR = 1.54), Vienna-CATS (HR = 1.91), CONKO (HR = 1.93), and PROTECHT (HR = 1.56) demonstrated a nearly twofold significantly increased risk of DP. Meanwhile, our previously reported HYPERCAN-VTE score showed the highest predictive value for DP (HR = 2.73).

## 4. Discussion

In this study, we evaluated the prognostic value of thromboinflammatory biomarkers in a large prospective observational cohort of patients with a new diagnosis of advanced-stage NSCLC, enrolled in the HYPERCAN study [25]. The ability to predict tumor progression is critically essential in the management and treatment of cancer. However, identifying predictive biomarkers and developing prognostic models requires robust data from extensive cohorts of prospective observational and multicenter studies specifically designed to identify these biomarkers, such as the HYPERCAN study.

The analyzed cohort consisted of 704 patients, of whom 79% were diagnosed with metastatic disease and 21% with locally advanced disease. The median age was 65, and 68% of the participants were male. A laboratory workup was conducted on samples collected from all patients at enrollment before initiating antitumor therapies. Within six months since study entry, the cumulative incidence of DP was 49%. Specifically, we observed progression in 342 patients, while 362 achieved DCR. These rates align with those reported in the literature [32,33,34,35,36].

Compared to patients who achieved DCR, the group of patients who progressed was characterized by a higher rate of individuals with metastatic disease and poorer ECOG performance status. These factors are well-known risk indicators for worse outcomes [7]. Additionally, the median leukocyte and platelet counts were significantly higher within the DP group than those without progression, along with lower hemoglobin levels. These hematological changes may be attributed to cancer-related inflammation, where elevated leukocyte counts are often observed, particularly with advanced tumor stages and metastatic disease [37]. No other clinical or hematological characteristic showed significant differences between the two groups.

A Cox analysis was conducted to determine prognostic factors for DP. Regarding the clinical-hematological variables at enrollment, metastatic disease (versus locally advanced disease), lower BMI and hemoglobin levels, and higher leukocyte count were found to be independent risk factors for DP. In contrast, radiotherapy appeared to offer a protective effect regarding the outcome. Interestingly, ECOG performance did not achieve statistical significance as a prognostic factor in our study, differing from the established literature, which identifies poor ECOG performance and low weight as well-documented factors linked to poor prognosis in patients with advanced-stage lung cancer [38,39,40]. This result can be attributed to the fact that only 8% of our patients had an ECOG performance score of 2, while the remaining patients had a score between 0 and 1. This distribution is consistent with our study protocol, which included patients eligible for antitumor treatment and projected to have a life expectancy exceeding three months [25].

Among thromboinflammatory biomarkers, patients who progressed were characterized by significantly higher baseline levels of FVIII, fibrinogen, hs-CRP, TAT complexes, and D-dimer compared to those who achieved DCR. The subsequent multivariable analysis, including the significant clinical and biomarker predictors, retained D-dimer and hs-CRP as the strongest predictors of the study outcome, independently of the stage (i.e., metastatic status). D-dimer has been systematically explored in relation to the prognosis of advanced-stage lung cancer patients, providing valuable insights into cancer-specific outcomes, including DP [21,41,42]. CRP, an acute-phase plasma protein produced in response to pro-inflammatory cytokines, has also been investigated as a prognostic marker for NSCLC patients [12,14,20,43,44]. Specifically, a recent study based on machine learning classifiers identified CRP as a strong predictor (HR = 3.7) of radiological progression [45]. However, both biomarkers have faced challenges in their validation for DP, as indicated by two large meta-analyses [21,46]. These challenges arise primarily from the variability of cut-off values that differentiate between low- and high-risk DP patients, as well as the fact that most studies supporting these results are retrospective with limited sample sizes.

In this line, the prospective framework of our study strengthens the existing evidence and provides a solid basis for validating these biomarkers in clinical practice. Our findings on the predictive value of hs-CRP and D-dimer aligned with previous research, encouraging us to assess their applicability through the creation of a prognostic model. This scoring system, based on the baseline levels of both biomarkers, aims to fully capture the prognostic significance of thromboinflammation. The model successfully classified patients’ risk into three distinct categories, each representing significantly different risks of developing DP, demonstrating the strongest predictive value for early DP at 3 and 6 months compared to the other timeframes analyzed; however, it can also be applied at 9 and 12 months. As presumed, the model’s predictive value surpassed that provided by each individual biomarker (i.e., hs-CRP and D-dimer evaluated through univariable analysis), demonstrating a synergistic effect. This may be because our model, by incorporating two non-collinear biomarkers, reflects a more complex biological background than a single biomarker, since cancer risk typically arises not from a single factor alone, but from the interaction of multiple biological pathways. To our knowledge, no existing studies explore the combined prognostic utility of hs-CRP and D-dimer specifically in NSCLC patients. The only related work, a prospective study of lung cancer patients followed for 24 months, integrated D-dimer and CRP into a broader biomarker model that also included the lymphocyte-to-monocyte ratio to predict DP and mortality [20]. While that model achieved a twofold increase in the discriminative power for patient prognosis stratification across quartiles (HR = 2.02), our model demonstrates a markedly superior threefold capability (HR = 2.91), highlighting its enhanced predictive strength. Beyond this, our scoring system is also user-friendly and does not rely on complex percentile calculations, making it practical for clinical use.

Several other prognostic models for NSCLC have been developed based on biomarkers related to inflammation and/or nutritional status, including peripheral blood cell count parameters, CRP, albumin, and BMI, such as the Prognostic Nutritional Index, the Advanced Lung Cancer Inflammation Index, and the Glasgow prognostic score [47,48,49]. These models have been applied in various scenarios concerning NSCLC, including early and advanced stages, among surgical resected patients, older age, and those undergoing adjuvant chemotherapy, immunotherapy, or targeted therapies [50,51,52,53]. However, the cut-off values used in these models have been extensively modified, resulting in a lack of standardization that limits their reliability [54].

Our study found that VTE was more common among patients who experienced DP, linking it to coagulation, inflammation, and cancer progression. This aligns with findings by Alexander M. et al., who noted a persistent hypercoagulable state in patients with early DP, indicated by increased fibrinogen, D-dimer, and platelet levels, alongside a thromboembolic event rate of 19% [55].

It is well accepted that identifying patients at a high risk of progression can enable early intervention with targeted therapies, thereby increasing the likelihood of treatment success and long-term survival [56]. In addition, unnecessary and potentially harmful treatments can be avoided, preserving the quality of life for low-risk patients [57]. According to our model, the 6-month cumulative incidence of DP was 30%, 57%, and 76% for the low, intermediate, and high-risk groups, respectively. With 76% DP, more aggressive and intensive treatments may be recommended to avoid toxicity in others with lower risks. Moreover, considering that among locally advanced patients, those who underwent surgery received 2 to 4 cycles of neoadjuvant chemotherapy, our score’s ability to predict outcomes within 3 months of starting treatment is a significant aspect, as it provides an early “red flag” of prognostic information. Standard practice often involves completing several cycles before re-evaluating the disease, which can delay necessary changes. If patients are predicted to have a worse outcome since enrollment according to our model, clinicians can swiftly consider and implement alternative strategies for potential treatments or closer monitoring, which may lead to improved outcomes.

These are not the first positive data emerging from the HYPERCAN study. Published data have demonstrated the capacity of some coagulation biomarkers for predicting DP and mortality in patients with metastatic colorectal cancer [58], early disease recurrence in surgically resected breast cancer patients [59,60,61], and VTE and mortality in metastatic NSCLC [31]. Together with these previous studies, the present analysis adds further importance to the study of hypercoagulability and inflammation in relation to cancer prognosis, beyond VTE.

However, we acknowledge that our study has some limitations. First, our scoring model yields an accuracy of 0.694, which might seem restrictive. Second, it requires validation in an external prospective cohort of NSCLC patients to confirm our findings. Another limitation is that most patients were enrolled before immune checkpoint inhibitors and targeted therapies became first-line treatments. Consequently, only a small number of patients receiving these contemporary interventions are included in the study. Nonetheless, the model consistently demonstrated prognostic utility, particularly beyond the 3-month mark post-treatment initiation. This compelling finding underscores the model’s potential for further validation within more contemporary patient cohorts. Testing with hs-CRP may raise some questions about the greater cost compared to testing with standard CRP. However, hs-CRP offers a significant advantage. Originally developed for cardiovascular risk assessment, hs-CRP can identify even subtle inflammation, making it a more sensitive tool than standard CRP tests [30]. This enhanced sensitivity may help in the early detection of inflammation and risk evaluation in patients without other signs of inflammation [62]. If research validates hs-CRP’s predictive capabilities, its routine use in certain cancer patient populations is justified, balancing potential benefits with costs and improving clinical outcomes. Our study has notable strengths. First, the model was developed using data from a large, multi-center, prospective study, which significantly enhances its reliability. Second, it is based on the well-established hs-CRP cut-offs, widely used in cardiovascular disease due to their standardization, along with the cut-off values for D-dimer from our prior publication, effectively representing the distribution of our patient population. Both biomarkers are readily available, and our scoring system is easy to understand and use. Furthermore, its robustness is assured through a 1000-fold bootstrapping process of internal validation, guaranteeing consistent predictions across diverse patient populations. Finally, the model demonstrates strong calibration, with predicted probabilities closely aligning with actual outcomes, instilling high confidence in DP predictions.

## 5. Conclusions

In conclusion, this prospective cohort study has developed a new, intuitive tool for quickly and accurately assessing early DP based on levels of hs-CRP and D-dimer, which are widely and frequently available and easily measurable biomarkers. If externally validated, this model can significantly improve the allocation of medical resources in managing advanced NSCLC, ensuring that patients receive the most effective care available. Research on tumor progression prediction is crucial for developing new, more effective treatment strategies. Understanding the molecular and cellular mechanisms that drive tumor progression enables the identification of new therapeutic targets and the development of targeted drugs. In summary, early recognition of cancer progression through an effective prognostic tool is vital for enhancing cancer management, personalizing treatment, improving outcomes, reducing toxicity, optimizing resource allocation, and fostering research.

## Figures and Tables

**Table 1:** General characteristics of the total cohort at enrollment according to 6-month DP and DCR.

	6-Month DCR (*n* = 362)	6-Month DP (*n* = 342)	*p*-Value
Male sex (*n*, %) Age (years, mean [SD]) BMI (kg/m^2^, mean [SD]) Metastatic disease (*n*, %) Locally advanced disease (*n*, %) ECOG (*n*, %) 0 1 2	237 (66) 65 (8.7) 25 (4.5) 264 (73) 98 (27) 190 (53) 138 (38) 9 (3)	243 (71) 66 (10.2) 24 (4.2) 297 (87) 45 (13) 120 (35) 159 (47) 43 (13)	0.066 0.061 0.121 <0.001 <0.001
Smoking (*n*, %) Active Former	118 (33) 160 (44)	119 (35) 155 (45)	0.367
CV risk factors ≥ 1 (*n*, %) Diabetes Hypertension Dyslipidemia Cardiopathy Stroke history	267 (74) 40 (11) 148 (41) 65 (18) 36 (10) 6 (2)	262 (77) 47 (14) 151 (44) 51 (15) 27 (8) 3 (1)	0.390 0.165 0.187 0.161 0.205 0.506
Histological subtypes (*n*, %) Squamous Adenocarcinoma Non-differentiated Other Non-classified	88 (24) 241 (67) 10 (3) 5 (1) 18 (5)	70 (21) 224 (66) 21 (6) 9 (3) 18 (5)	0.060
Blood Count (median [IQR]) Leukocyte, 109/L Hemoglobin, g/dL Hematocrit, % Platelets, 109/L	8.8 (6.9–11.1) 13.4 (12.3–14.5) 40.5 (37.8–43.3) 272 (213–335)	9.5 (7.3–12.7) 13.0 (11.8–14.1) 39.7 (35.9–42.3) 289 (218–379)	0.001 0.001 0.001 0.042
Chemotherapy (*n*, %) Carboplatin-Paclitaxel Carboplatin-Pemetrexed Carboplatin-Gemcitabine Carboplatin-Vinorelbine Taxane Gemcitabine Vinorelbine Other Immunotherapy (*n*, %) * Target therapy (*n*, %) * Radiotherapy (*n*, %) Radical Palliative	362 (100) 20 (6) 159 (44) 105 (29) 21 (6) 2 (1) 15 (4) 6 (2) 34 (9) 18 (5) 13 (4) 195 (54) 52 (14) 145 (40)	342 (100) 10 (3) 157 (46) 94 (26) 13 (4) 0 (0) 32 (9) 21 (6) 15 (4) 43 (13) 17 (5) 153 (45) 16 (5) 135 (40)	0.122 <0.001

Categorical data are presented as numbers (percentages) and shown for patients who have developed disease progression or remained disease-progression-free within 6 months from enrollment. Age and body mass index are expressed as means with standard deviations. Blood cell count data are presented as median with interquartile range. * Immunotherapy and target therapy were applied in combination with systemic chemotherapy. DP: disease progression, DCR: disease control rate, SD: standard deviation, BMI: body mass index, ECOG: Eastern Cooperative Oncology Group performance status, CV: cardiovascular, IQR: interquartile range.

**Table 2:** Univariable and multivariable analysis of the effect of clinical and laboratory variables effect on 6-month disease progression.

	Univariable Analysis		Multivariable Analysis	
Variables	HR (95% CI)	*p*-Value	HR (95% CI)	*p*-Value
**Age, years**	1.008 (0.997–1.020)	0.166		
**Male sex**	0.890 (0.754–1.051)	0.171		
**Metastatic status**	1.644 (1.316–2.052)	<0.001		
**BMI**	0.973 (0.949–0.999)	0.038		
**CV risk factors**	1.000 (0.837–1.195)	0.997		
**Adenocarcinoma vs. squamous**	1.129 (0.934–1.365)	0.209		
**ECOG = 2**	1.114 (0.919–1.349)	0.271		
**Radical vs. palliative radiotherapy**	0.622 (0.522–0.741)	<0.001		
**Leukocytes, 10^9^/L**	1.032 (1.018–1.045)	<0.001		
**Hemoglobin, g/dL**	0.894 (0.841–0.949)	<0.001		
**Platelets, 10^9^/L**	1.001 (1.000–1.002)	0.051		
**hs-CRP, mg/dL**	1.070 (1.050–1.089)	<0.001	1.083 (1.055–1.111)	<0.001
**FVIII, %**	1.003 (1.002–1.005)	<0.001		
**Fibrinogen, mg/dL**	1.001 (1.000–1.001)	<0.001		
**TAT, µg/L**	1.122 (1.027–1.225)	0.011		
**F1+2, pmol/L**	1.000 (1.000–1.001)	0.223		
**D-dimer, µg/mL**	1.001 (1.000–1.002)	<0.001	1.001 (1.000–1.002)	0.018

Univariable Cox proportional hazard model analysis evaluating 6-month disease progression. The multivariable model considered the significant (*p* < 0.05) variables identified in the univariable analysis. BMI: body mass index, CV: cardiovascular, ECOG: Eastern Cooperative Oncology Group performance status, hs-CRP: high sensitivity C-reactive protein, FVIII: factor VIII, TAT: thrombin-antithrombin complex, F1+2: prothrombin fragment 1+2.

**Table 3:** Thromboinflammatory-based model for DP.

hs-CRP (mg/dL)	Points
<1.0	1
1.0–3.0	2
>3.0	3
**D-dimer (µg/mL)**	
<0.5	0
0.5–1.5	1
>1.5–4.0	2
>4.0	3

1–2: low risk; 3–4: intermediate risk; 5–6: high risk.

## Data Availability

The data presented in this study are available on request from the corresponding author.

## Supplementary Materials

The following supporting information can be downloaded at: https://www.mdpi.com/article/10.3390/cancers17121932/s1, Figure S1: Thromboinflammatory biomarkers according to complete response (CR), partial response (PR), and disease progression (DP); Table S1: Predictive value of the developed model in different sub-cohorts of patients; Table S2: Predictive value of models developed for venous thromboembolism (VTE) applied for disease progression (DP). The complete list of the HYPERCAN collaborators is listed in the supplemental materials.
