# Supplementary material for: Thromboinflammatory Biomarkers Are Early Predictors of Disease Progression in Non-Small Cell Lung Cancer Patients
Source: Cancers (Basel). 2025 Jun 10;17(12):1932. doi: 10.3390/cancers17121932 (PMC12190336; doi:10.3390/cancers17121932)
Supplement: Supplementary file 1 [file cancers-17-01932-s001.zip › Supplemental material.pdf]

‡ **Colaborators:** The members of the HYPERCAN Study (by centers, all in Italy) are the following: Coordinating Center: Immunohematology and Transfusion Medicine, Hospital Papa Giovanni XXIII, Bergamo: Marchetti Marina, Falanga Anna, Gomez-Rosas Patricia, Testa Maria, Motta Alessia, Tartari Carmen Julia, Russo Laura, Bolognini Silvia, Ticozzi Chiara, Debora Romeo, Schieppati Francesca, Luca Barcella. Participants: Oncology Unit, IRCCS Humanitas Research Hospital, Rozzano Milan: Masci Giovanna, Santoro Armando. Oncology Unit, IRCCS National Cancer Institute, Milan: De Braud Filippo, Celio Luigi. Oncology Unit, Hospital Papa Giovanni XXIII, Bergamo: Tondini Carlo, Labianca Roberto. Oncology Unit, Hospital San Filippo Neri, Rome: Gasparini Giampietro, Sarmiento Roberta. Oncology Unit, Hospital Treviglio-Caravaggio, Treviglio: Petrelli Fausto. Medical oncology and Internal Medicine, University Vita-Salute San Raffaele, Milan: D'Alessio Andrea. Oncology Unit, IRCCS Cancer Institute Giovanni Paolo II, Bari: Giuliani Francesco.
