# Supplementary material for: Thromboinflammatory Biomarkers Are Early Predictors of Disease Progression in Non-Small Cell Lung Cancer Patients
Source: Cancers (Basel). 2025 Jun 10;17(12):1932. doi: 10.3390/cancers17121932 (PMC12190336; doi:10.3390/cancers17121932)
Supplement: Supplementary file 1 [file cancers-17-01932-s001.zip › Supplemental Table 1.pdf]

Table S1. Model applied to different sub-cohorts

	3-month DP		6-month DP		9-month DP		12-month DP	
Sub-cohort	**HR (95%CI)**	**p-value**	**HR (95%CI)**	**p-value**	**HR (95%CI)**	**p-value**	**HR (95%CI)**	**p-value**
Metastatic (n=561)	2.257 (1.821-2.797)	<0.001	2.022 (1.711-2.390)	<0.001	1.685 (1.455-1.951)	<0.001	1.588 (1.381-1.825)	<0.001
Locally advanced (n=143)	2.368 (1.096-6.347)	0.030	2.420 (1.366-4.365)	0.007	2.099 (1.310-3.364)	0.002	1.811 (1.183-2.774)	0.006
Patients treated with immunotherapy or target therapy (n=91)	2.177 (0.973-4.872)	0.058	2.396 (1.154-4.973)	0.019	1.973 (1.062-3.668)	0.032	1.944 (1.062-3.556)	0.031
