# Supplementary material for: Thromboinflammatory Biomarkers Are Early Predictors of Disease Progression in Non-Small Cell Lung Cancer Patients
Source: Cancers (Basel). 2025 Jun 10;17(12):1932. doi: 10.3390/cancers17121932 (PMC12190336; doi:10.3390/cancers17121932)
Supplement: Supplementary file 1 [file cancers-17-01932-s001.zip › Supplemental Table 2.pdf]

Table S2. Predictive value of models developed for VTE applied for DP

	6-month DP	
**Scores for VTE**	**HR (95% CI)**	**p-value**
HYPERCAN-VTE	2.727 (2.174-3.422)	<0.001
KRS	1.536 (1.235-1.911)	<0.001
PROTECHT	1.559 (1.252-1940)	<0.001
CONKO	1.927 (1.482-2.506)	<0.001
New-Vienna CATS	1.908 (1.480-2.460)	<0.001
COMPASS-CAT	1.095 (0.797-1.504)	0.575
